# Localisation of Nursery Areas Based on Comparative Analyses of the Horizontal and Vertical Distribution Patterns of Juvenile Baltic Cod (*Gadus morhua*)

**DOI:** 10.1371/journal.pone.0070668

**Published:** 2013-08-14

**Authors:** J. Rasmus Nielsen, Bo Lundgren, Kasper Kristensen, Francois Bastardie

**Affiliations:** 1 Technical University of Denmark, National Institute of Aquatic Resources, Charlottenlund, Denmark; 2 Technical University of Denmark, National Institute of Aquatic Resources, Hirtshals, Denmark; University of Canterbury, New Zealand

## Abstract

Knowledge of the spatial distribution of juvenile cod is essential for obtaining precise recruitment data to conduct sustainable management of the eastern and western Baltic cod stocks. In this study, the horizontal and vertical distribution and density patterns of settled juvenile 0- and 1-group Baltic cod are determined, and their nursery areas are localised according to the environmental factors affecting them. Comparative statistical analyses of biological, hydrographic and hydroacoustic data are carried out based on standard ICES demersal trawl surveys and special integrated trawl and acoustic research surveys. Horizontal distribution maps for the 2001–2010 cohorts of juvenile cod are further generated by applying a statistical log-Gaussian Cox process model to the standard trawl survey data. The analyses indicate size-dependent horizontal and distinct vertical and diurnal distribution patterns related to the seabed topography, water layer depth, and the presence of hydrographic frontal zones (pycnoclines) as well as intraspecific patterns in relation to the presence of adult cod. The extent of the nursery areas also depends on the cod year class strength. Juvenile cod (≥3 cm) are present in all areas of the central Baltic Sea (CBS), showing broad dispersal. However, their highest density in the Baltic Basins is found at localities with a 40–70 m bottom depth in waters with oxygen concentrations above 2 ml O_2_.l^−1^ and temperatures above 5°C. The smallest juveniles are also found in deep sea localities down to a 100 m depth and at oxygen concentrations between 2–4 ml O_2_.l^−1^. The vertical, diurnally stratified and repeated trawling and hydroacoustic target strength-depth distributions obtained from the special surveys show juvenile cod concentrations in frontal zone water layers (pycnocline). However, the analyses indicate that in the CBS, juvenile cod of all sizes do not appear to aggregate in dense schooling patterns, which differs from what has been reported from the North Sea.

## Introduction

The changes in hydrographic features and potential changes in cod spawning areas and nursery ground locations over time, together with the resulting recruitment variability and possibly different recruitment regimes [1], [2], [3], [4], [5], heavy exploitation by fisheries and likely changes in migration at age between the two Baltic cod stocks [1], [6], [7] complicate the long-term management of the stocks [8], [9], [10]. This situation should be seen in the light of the fact that the adult cod in the eastern Baltic Sea are distributed at one of their environmental limits regarding salinity and oxygen tolerance [11], [12] and that their abundance has changed considerably in historical times as a result of variations in the environment [1], [3], [13], [4], [5], [14]. Extensive long-term fluctuations in stock recruitment have proven to depend on climate-driven hydrographic conditions and regime shifts [15], [1], [2], [3], [16], [4], [6], [17], [7]. All of these factors call for deeper investigations of juvenile cod distribution patterns and variations in the central Baltic Sea.

In general, there is a gap in the available scientific knowledge regarding the biology and population dynamics of 0- and 1-group settled juvenile Baltic cod (*Gadus morhua*) [18], [19], [3], [14], [7], [17]. An important reason for this gap is the lack of adequate coverage in research surveys and the fact that these life stages are not caught in commercial fisheries [20], [7], [21]. The processes and pressures associated with these life stages and the variability in their distribution and abundance patterns as well as their nursery grounds are not well documented in terms of Baltic cod life cycle dynamics [22], [3], [1], [7].

In 2001, the EU research project ISDBITS (see references) introduced a completely revised international standardized BITS survey (ICES Baltic International Trawl Survey) [23], [20], [21] with the aim to introduce new demersal survey gear and a new stratified random sampling survey design, expanding seasonal and geographical sampling to obtain better coverage of cod distribution areas in all life stages. In particular, a focus is concentrated on more efficiently covering of the settled stages of juvenile cod by increasing the survey fishing power for these life stages [23], [20]. Accordingly, the quality of the survey indices has increased, and more recruitment and abundance at age data at a higher coverage have been obtained for use in ICES Baltic cod stock assessments for management purposes and in research on population dynamic [7], [21].

In the traditional BITS, the participating nations used very different trawls, usually equipped with large bobbins, causing smaller cod to escape under the footrope [24]. ISDBITS employs new internationally standardized survey trawls of the commercial TV3 type mounted with rubber disc bottom gear exhibiting close seabed contact and being robust to the CBS bottom topography [23], [20], [21]. Furthermore, statistically robust and standardized inter-calibration methods to link old and new survey data time series have been developed and implemented to estimate the trawl survey efficiency and fishing power (and selectivity) as well as to link indices obtained using different sizes of the new standard gear [20], [21]. After 12 years of implementation of this new survey design, the understanding of juvenile cod distribution patterns and of the spatio-temporal patterns in recruitment dynamics can now be improved via thorough analyses of the obtained BITS data.

Such analyses should contribute to validating the predictions of the advanced 3D- hydrodynamic drift model currently applied in the Baltic Sea [22], [3], where the transport patterns for eastern Baltic cod eggs and larvae according to the spawning area and time have been simulated for the periods 1986–99 and 1979–2004. The model predicts which habitats show a high probability of successful settling of early demersal stage juvenile cod, depending on the oxygen saturation. The predicted habitats are located in the shallow-water areas at the edges of the basins (40–60 m bottom depth) down to where the halocline hits the bottom, while the settlement probability in the deeper central parts of the basins is low due to the minimum oxygen requirements for successful settling. These predictions are to be verified based on the present updated observed distributions from pelagic and demersal trawl surveys because previous BITS, Baltic hydroacoustic research surveys and commercial fishery data [7], [21] have not covered juveniles adequately.

Among the explanatory factors, interspecific relationships and potential intraspecific density dependence may play a role in the distribution patterns of Baltic cod in relation to other Baltic fish species, but neither factor is well understood [25], [17]. There is temporal variation in biological interactions due to predation by cod and food availability related to prey stocks such as sprat (*Sprattus sprattus*) and herring (*Clupea harengus*) in the Baltic, and size dependent predation can be central in relation to cod recruitment because cannibalism has been documented as an impacting factor in certain periods [26], [27], [28], [18], [14], [29], [17]. The levels of cannibalism are dependent on the abundance of juveniles and larger cod predators, their overlap in distribution, and the availability of alternative prey items for larger cod, such as sprat and herring [30], [18], [29]. Additionally, in the western Baltic Sea, there are competing gadoid predators in the form of whiting (*Merlangius merlangus*) [7]. Consequently, the present investigation of juvenile cod distribution dynamics in relation to cod predators is relevant.

Kristensen [31] and Lewy and Kristensen [32] estimated North Sea cod distribution patterns with their Log-Gaussian Cox Process (LGCP) model, determining correlations in densities using a statistical approach based on spatial correlations between observations from surveys and fisheries according to age. A length-based stochastic model of single-species stock dynamics including densities [33] has been applied for Baltic cod based exclusively on survey data; however, this model is not spatially explicit. An extension of the LGCP model was applied to mackerel (*Scombrus scombrus*) larvae survey data [34] based on additional temporal co-variance in spatial distributions. The LGCP model provides densities with high resolution in time and space for survey data. In the present study, a similar extension of the LGCP model is applied to the BITS data, but with a further extension in the form of following individual cohorts to describe the distribution and density patterns of settled 0- and 1-group Baltic cod.

The below 0-hypotheses (where the hypotheses are not mutually independent) regarding the settled Baltic juvenile cod distribution, density and abundance patterns are tested in the present study based on the new, revised BITS data, with a new survey design and recent improved survey data analysis methods. The analyses mainly cover the life stages before recruitment to the fishery, and in the ICES stock assessments.

H01: Settled juvenile Baltic cod are only present in shallower (more oxygen saturated) areas down to a 60 m depth in the Baltic Sea, e.g., at the edges of the Baltic basins; i.e., juvenile Baltic cod exhibit distinct and limited geographical nursery areas, without considerable variation over years.

H02: Settled juvenile Baltic cod aggregate in dense schools and show schooling behaviour, as observed for juvenile cod in the North Sea.

H03: Settled stages of juvenile Baltic cod do not show distinct vertical distribution patterns related to hydrographic vertical zoning.

H04: There is no dependency of the occurrence of settled juvenile cod in relation to larger cod (potential predator size group) or of their distribution in relation to year class strength.

## Materials and Methods

### Survey coverage and stratification

**Figure 1:**
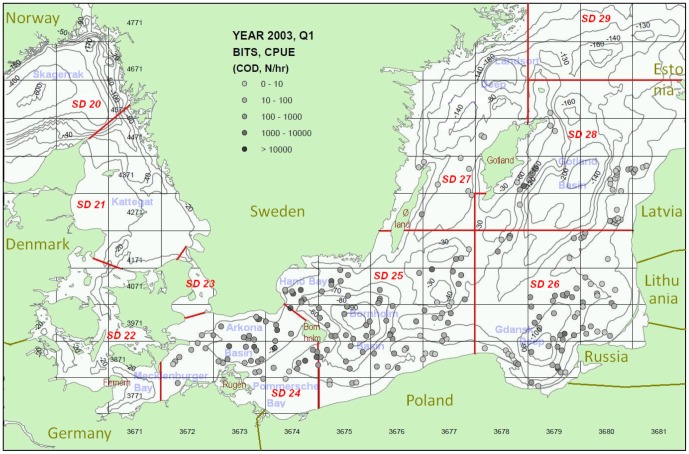
Investigation area for juvenile cod distribution and coverage of the stratified random and standardized ICES BITS trawl survey with new survey design according to Nielsen *et al.* (2001) and Lewy *et al.* (2004) including station specific catch rates of cod (example from the 1^st^ quarter 2003 survey).

**Figure 2:**
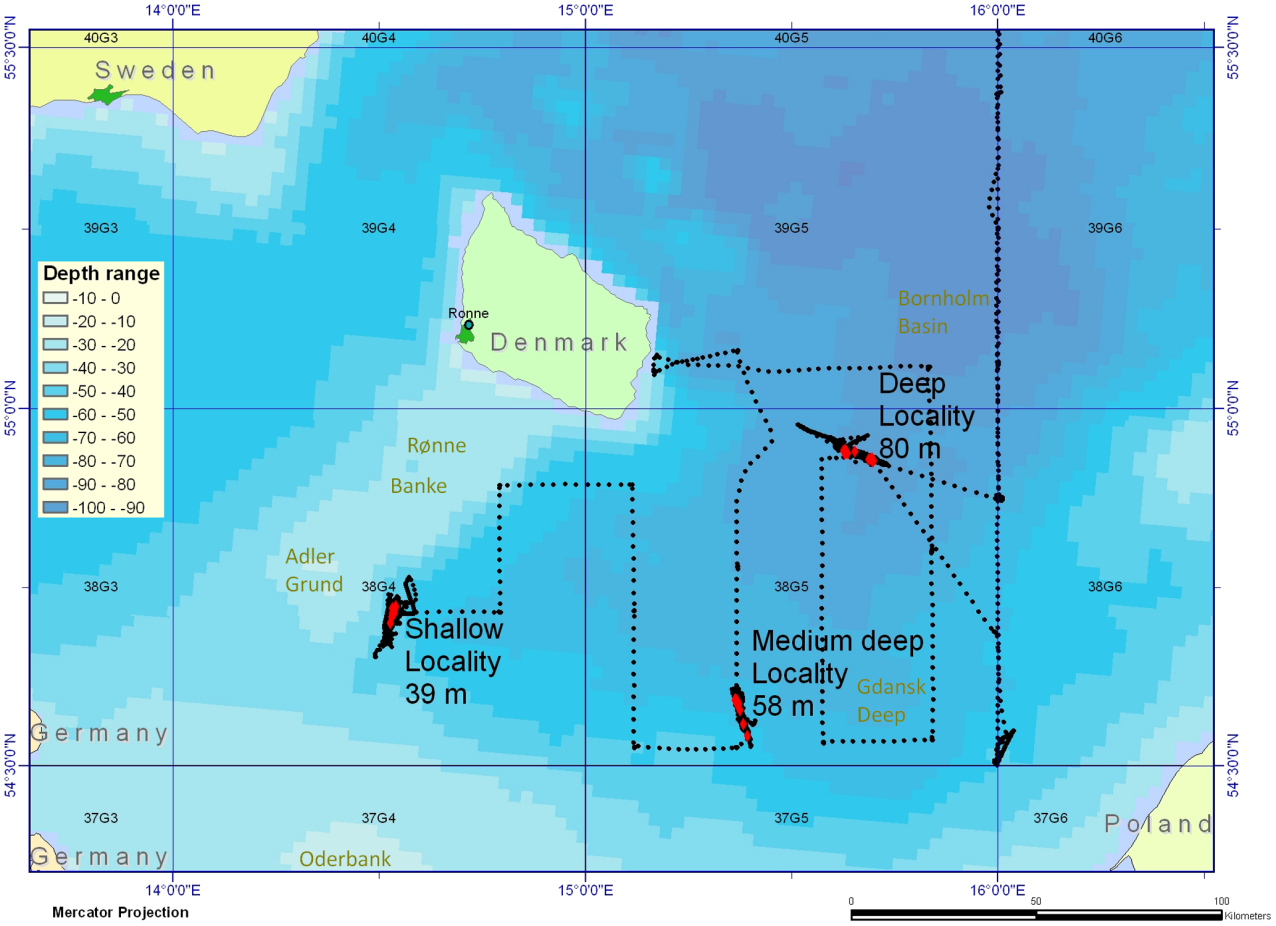
Coverage and topography of the selected stations of the 3 types of central Baltic localities (shallow Bank, medium deep, and deep basin locality) investigated by intensive and combined trawl, hydrographic and hydroacoustic transects between localities (including 2 days and 2 nights continuous sampling at each locality) during the specialized surveying in 1998.

The BITS survey manual [21] describes the revised (2001 and thereafter) standardized and stratified random BITS TV3-bottom-trawl surveying and sampling methods, including the format of the BITS data which are available at the ICES DATRAS database (www.ices.dk). The BITS survey is stratified according to ICES subdivision (SD) and depth. The geographical coverage of the BITS cod trawl sampling data analysed in this study corresponds to ICES SDs 24–29, which represent the different Baltic basins and deeps which are important ecosystem units in cod spawning and recruitment ecology, i.e. the Arkona Basin (SD24), the Bornholm Basin area and around Bornholm, and the Bank areas SW of Bornholm, as well as the Hanö Bay (SD25), the Gotland Basin area (SD28), and the Gdansk Deep area (SD26) (see example in Fig. 1). Additional trawl sampling was performed during specialised integrated multi-task trawl and acoustic juvenile cod distribution surveys repeated in 1995, 1997 and 1998 (Table 1; [35]) as a part of the EU-FP4-AIR2-94-1226 Baltic Cod Recruitment Project. The repeated surveys were a part of more than 14 surveys (1994–1998) under the project with broad sampling of biological and physical-chemical oceanographic and acoustic data (Fig. 2) covering different seasons and areas of the CBS. These surveys targeted the early life stages of cod, including the eggs, larvae, and 0- and 1-group metamorphosed juveniles. For the repeated surveys (1995, 1997, 1998) selected areas of the Baltic Proper (SDs25–26) were subdivided into three main types of cod habitats according to physical and biological environmental conditions and bottom depths: Area 1: A shallow-water area southwest of Bornholm and the Bornholm Basin at the Rønne Bank, Adler Ground, and Oder Bank (SW Baltic Sea); Area 2: A medium-depth area in the Gdansk Deep area located southeast of the Bornholm Basin (SE Baltic Sea); and Area 3: A deep-sea area in the Bornholm Basin area east of Bornholm and north of the other areas. (Fig. 2; Table 1). Here, he monitoring covered trawl sampling, the sea bed topography, and hydrographic features including variation in vertical physical frontal zones (the presence and depth of the pycnocline) based on CTD (Conductivity Temperature Depth Profiler) measurements. The specialized survey data are stored in the DTU Aqua databases and can be made available through DTU Aqua IT Management (www.aqua.dtu.dk). The benthic biological habitats were also characterised according to the density patterns of major food items for juvenile cod, e.g., the abundance of mysids (*Mysidae*), measured via hydroacoustic methods.

**Table 1:** Division of fishing stations at the repeated specialized surveys with small meshed EXPO trawl by locality, time of day and hauling layer during at the R/V Dana winter surveys December 1998 (DS1698), January 1997 (DS0197), and December 1995 (DS1295).

Stratum	Time	Layer DS1698	Stations DS1698	Layer DS0197	Stations DS0197	Layer DS1295	Stations DS1295
Shallow water locality	Night	Bottom	10, 32	Full layer	7		
54.43N 14.32E		Surface	(7), (8), 29, 36, 38				
	Day	Bottom	21, 24, 45			Full layer	81
		Surface	18, 42				
Medium deep locality	Night	Bottom	59, 82	Full layer	11, 13	Full layer	107, 109, 110, 111
54.35N 15.22E		Medium layer	(58), 62, 80, 89				
		Surface	57, 61, 78				
	Day	Bottom	70, 73, 99, 103	Full layer	78, 80, 81, 82, 84		
		Medium layer	68, 97				
		Surface	66, 95				
Deep sea locality	Night	Bottom	128, 169				
54.56N 15.40E		Medium layer	126, 170				
		Surface	124				
	Day	Bottom	117, 158				
		Medium layer	115, 160				
		Surface	113, 119, 161				

### Biological trawl sampling

The fish sampling was designed according to standard procedures presented in the BITS Manual [21]. During the 1998 specialised survey in particular, standardized and depth-stratified fishery sampling was performed through repeated day and night hauls at the selected localities (Table 1; Fig. 2) covering 2 full days and 2 nights per locality per survey. This sampling was performed with a large, combined demersal and pelagic EXPO trawl equipped with small bobbins and using a pelagic young fish trawl (IYGPT), both with a stretched codend mesh size of 16 mm, in addition to a smaller-meshed pelagic MIK ring trawl to a lesser extent. Nearly all hauls performed with the EXPO and IYGPT trawls were double oblique (V-shaped) hauls covering a specific targeted vertical water layer (Table 1) as well as the sea bottom, when performing targeted bottom hauls. As such, isolated demersal and pelagic hauls in specific water layers were conducted to identify juvenile cod vertical distribution patterns. The active fishing time with the EXPO trawl was usually 40 minutes, of which 25 minutes was devoted to trawling in the targeted vertical water layer. The hauling speed was between 3.4 and 4.1 knots, typically ranging from 3.8–3.9 knots. The trawl gap varied from 6 m, at the bottom, to 8 m, when pelagic, and the trawl width was between 90 and 105 m (typically 100 m). The details of the BITS and specialised surveying procedures are shown in Figures 1–2 and Table 1. In general, cod were not caught in the pelagic IYGPT and MIK trawls during the specialised surveys, so only the EXPO activities are shown in Table 1.

### Hydrographic CTD recording

To localise the pycnocline and determine the near-bottom salinity, temperature, and oxygen concentration at the trawling localities a vertical CTD profile of the water column structure was obtained for each trawling event using a SEABIRD SBE 911+ model CTD with standard probes for pressure, conductivity, temperature, and oxygen (Table S1 in File S1). The profiles covered the entire vertical water column, including the near-bottom layer. The CTD probes were calibrated before each survey, and cross-checking was performed by taking salinity and oxygen water samples using a GO rosette sampler during up-casts. Salinity of the water samples was measured with a Guildline Portasal 8410A. The oxygen profiles were corrected by linear regression based on results from Winkler titration of the oxygen water samples.

### Hydroacoustic data recording

Acoustic data were collected using the Simrad EY500 portable scientific 38 kHz split beam echosounder system (version 5.0 [36]) with an ES38-B-type hull-mounted transducer placed at 6 m depth below the sea surface. The parameter settings are shown in the Table S1 in File S1. An external power supply was employed to increase the pulse power to 987 W to improve the signal-noise ratio. The parameters for sound speed and absorption coefficient were set to 1450 m s^−1^ and 4 dB km^−1^, respectively, to account for the average values below the transducer derived from the salinity and temperature measurements. The system was calibrated before each survey according to the standard copper sphere technique [37], [38], [39], [40], [41]. Transects of raw split-beam data were collected along the entire hauling transect at all trawl stations during the specialised surveys to obtain spatially overlapping and activity-specific acoustic profiles that were directly comparable to the trawl sampling data (Fig. 2). Supplementary acoustic data collection (Fig. 2) was performed between the trawl stations. The raw data were analysed with the Echoview Version 4.6 software. The original target strength (TS) values produced by the echosounder were not used. Instead, the targets were redetected and the TS values recalculated using the Single-targets Method 1 operator in Echoview (http://www.echoview.com/support/echoview-technical-manual). This operator applies an improved version of the algorithm implemented in the Simrad EK500 software to detect single targets from echo data [42], [43], [44], [45]. The analyses were performed for the water layer from 3 m below the transducer to 0.5 m above the bottom echo.

### Comparative data analysis of juvenile cod distribution patterns

First, a size-based generalised linear model (GLM) analysis was applied assuming negative binomial distributions and over-dispersion [46]. Then, the statistical LGCP correlation model was applied on the same data to determine the high-resolution density patterns of the 0- and 1-group cod cohorts through spatial and temporal correlations between survey observations based on previously described methodology [34], [31], [32]. In the present application, the LGCP model was further advanced to also follow the correlations within individual cod cohorts. The output from these statistical analyses of the density and distribution patterns was compared with the ICES assessment working group [7] cod year class strength estimates. Finally, the data analysis comprises an integrated analysis of the combined trawl catch data (Table 2) and hydroacoustic data from the specialised surveys, with a focus on the 1998 sampling targeting the vertical distribution of juvenile cod.

**Table 2:** Year class strength of eastern Baltic cod by year.

Year/Year Class	Inflow Strength into the Baltic	Recruitment age 2, XSA, ICES WGBFAS	Recruitment age 2, SAM, ICES WGBFAS	0-group BITS Q4 index from ICES DATRAS	1-group BITS Q1 index from ICES DATRAS	2-group BITS Q1 index from ICES DATRAS	Year Class Strength
2000	Weak	122472	116891	13783	42662	271924	(Weak)
2001	Weak	112745	95130	7516	38347	61747	Weak
2002	Weak	115077	99808	2470	10267	74005	Weak
2003	Strong	164235	138552	57669	83266	238898	Strong
2004	Weak	131041	129444	10838	58983	110839	Weak
2005	Weak	143846	140646	9509	6559	160561	Weak
2006	Weak	158464	152665	22907	58475	297516	Strong
2007	Weak	161770	156217	9867	36435	213619	(Strong)
2008	Weak	192503	177549	14774	28146	173658	(Strong)
2009	Weak	205390	192914	8107	7444	160703	(Strong)
2010	Weak		184795	3090	10105	171725	(Strong)
2011	Strong			11694	6788		?

Recruitment at age 2 in thousands from ICES WGBFAS, and CPUE index at age 0 and 1 by quarter from the ICES BITS DATRAS Database from spring 2012. Sources: ICES WGBFAS [5]; ICES DATRAS Database July 2012 (www.ices.dk). In the period 2000–2005 there were strong inflow events in spring 2003 and Nov/Dec 2011 of saline and oxygenated North Sea water into the Baltic while the inflow was weak in the rest of the period. Sources: [23], [49], [50], [24], [51], http://www.smhi.se/en/News-archive/improved-oxygen-conditions-in-the-baltic-deep-water-1.21801#bottom.

### Generalised linear model statistical data analysis

Prior to analysis, the raw BITS catch data for each haul were grouped into length groups and classified according to the year, quarter of the year, area (locality), and seabed depth (Eq. 1):
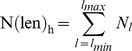
(1)where 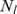 is the number of cod caught per haul per 1 cm length group, 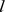, and N(len)_h_ is the number of cod caught per haul (h; by survey) per length group. The group class length is 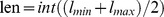, where 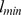 is the smallest length group, and 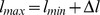 is the largest and where 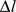-values of 5 or 10 cm were used. The raw catch data per haul were used as a proxy for the catch per unit of effort (CPUE) because the standard haul duration was 30 min, and hauls with a duration of less than 25 min or more than 35 min were excluded from the analyses (only few hauls). The data were not standardized to 1-hour hauls because the negative binomial distribution (see below) was not well suited to handle such standardisation. In the GLM analyses, the dependent variable CPUE was categorised into 5 cm cod length groups (Eq. 1; Table 3; Table S2 in File S1). In some instances, the consistency in length dependency was tested with an alternative 10 cm length stratification of the juvenile cod. The investigated area was surveyed using standard TV3 trawls of two different sizes in different ICES subdivisions (a small TV3 in SDs24–25 and a large TV3 in SDs25–29). Gear calibration was performed [23], [20], and conversion factors between the two trawls according to species and fish length groups were previously reported [20]. For cod, CPUE values obtained with the small TV3 trawl were converted to equivalent CPUE values for the large TV3 trawl with the following conversion factors: length less than 10 cm, 1/1.68; 10–15 cm (10 cm incl. and 15 cm excl.), 1/1.06; 15–20 cm, 1/1.15; 20–25 cm, 1/0.98; 25–30 cm, 1/0.91; 30–35 cm, 1/0.80; 35–40 cm, 1/0.81; 40–45 cm, 1/0.61; 45–50 cm, 1/1.12; 50 cm and larger, 1/1.29.

**Table 3:** Generalized linear model maximum likelihood parameter estimates of CPUE by using the SAS GENMOD Procedure for the statistical model 1 (Eq. 2), and the negative binomial dispersion parameter estimated by maximum likelihood.

				Wald 95%		Wald				Wald 95%		Wald				Wald 95%		Wald				Wald 95%		Wald	
Statistic		Estimate	SE	Confidence Limits		Chi-Sq	Pr>ChiSq	Estimate	SE	Confidence Limits		Chi-Sq	Pr>ChiSq	Estimate	SE	Confidence Limits		Chi-Sq	Pr>ChiSq	Estimate	SE	Confidence Limits		Chi-Sq	Pr>ChiSq
Length Group		3 cm	3 cm	3 cm	3 cm	3 cm	3 cm	8 cm	8 cm	8 cm	8 cm	8 cm	8 cm	13 cm	13 cm	13 cm	13 cm	13 cm	13 cm	18 cm	18 cm	18 cm	18 cm	18 cm	18 cm
**Parameter**																									
**Intercept**		−1.6816	0.5891	−2.8363	−0.5270	8.15	0.0043	0.4981	0.2635	−0.0183	1.0146	3.57	0.0587	−0.4266	0.2503	−0.9173	0.0640	2.90	0.0883	2.0371	0.2162	1.6134	2.4608	88.79	<,0001
**Year**	**2001**	0.7497	0.4895	−0.2098	1.7092	2.35	0.1257	0.8443	0.2202	0.4127	1.2758	14.70	0.0001	1.4525	0.2086	1.0436	1.8614	48.46	<,0001	0.1684	0.1901	−0.2042	0.5410	0.78	0.3757
**Year**	**2002**	−0.0285	0.5226	−1.0528	0.9957	0.00	0.9565	0.6211	0.2245	0.1810	1.0612	7.65	0.0057	0.3762	0.2166	−0.0483	0.8007	3.02	0.0824	0.4831	0.1870	0.1165	0.8496	6.67	0.0098
**Year**	**2003**	0.8708	0.4955	−0.1004	1.8420	3.09	0.0789	1.3507	0.2290	0.9019	1.7996	34.79	<,0001	1.2338	0.2222	0.7984	1.6692	30.84	<,0001	0.6233	0.2040	0.2234	1.0232	9.33	0.0022
**Year**	**2004**	1.1931	0.4876	0.2374	2.1487	5.99	0.0144	1.0436	0.2220	0.6085	1.4786	22.10	<,0001	1.8225	0.2123	1.4065	2.2386	73.72	<,0001	1.2371	0.1874	0.8699	1.6044	43.59	<,0001
**Year**	**2005**	0.1071	0.4957	−0.8645	1.0787	0.05	0.8289	−0.4994	0.2220	−0.9345	−0.0643	5.06	0.0245	0.0149	0.2126	−0.4017	0.4315	0.00	0.9442	0.7841	0.1799	0.4315	1.1366	19.00	<,0001
**Year**	**2006**	0.1763	0.4997	−0.8031	1.1557	0.12	0.7243	0.1368	0.2284	−0.3109	0.5844	0.36	0.5493	−0.0174	0.2174	−0.4435	0.4087	0.01	0.9362	−0.1406	0.1907	−0.5144	0.2333	0.54	0.4611
**Year**	**2007**	1.2457	0.4842	0.2967	2.1948	6.62	0.0101	0.2039	0.2228	−0.2327	0.6405	0.84	0.3601	1.1825	0.2097	0.7714	1.5935	31.79	<,0001	0.3454	0.1837	−0.0147	0.7054	3.53	0.0601
**Year**	**2008**	0.8620	0.4966	−0.1112	1.8353	3.01	0.0826	0.5231	0.2276	0.0770	0.9692	5.28	0.0215	0.5849	0.2140	0.1655	1.0044	7.47	0.0063	0.5195	0.1866	0.1538	0.8852	7.75	0.0054
**Year**	**2009**	−0.2615	0.5002	−1.2419	0.7189	0.27	0.6011	0.3050	0.2230	−0.1321	0.7421	1.87	0.1714	0.3088	0.2109	−0.1045	0.7221	2.14	0.1431	0.1893	0.1880	−0.1792	0.5577	1.01	0.3140
**Year**	**2010**	−0.5025	0.5242	−1.5300	0.5249	0.92	0.3377	−0.5842	0.2333	−1.0414	−0.1270	6.27	0.0123	0.6323	0.2238	0.1935	1.0710	7.98	0.0047	0.0943	0.1861	−0.2704	0.4589	0.26	0.6123
**Year**	**2011**	0.0587	0.5014	−0.9240	1.0414	0.01	0.9068	−0.4109	0.2323	−0.8661	0.0444	3.13	0.0769	−0.7875	0.2238	−1.2262	−0.3489	12.38	0.0004	−1.3631	0.1923	−1.7400	−0.9862	50.24	<,0001
**Year**	**2012**	0.0000	0.0000	0.0000	0.0000			0.0000	0.0000	0.0000	0.0000			0.0000	0.0000	0.0000	0.0000			0.0000	0.0000	0.0000	0.0000		
**Quarter**	**1**	−1.6659	0.1512	−1.9623	−1.3695	121.33	<,0001	0.1994	0.0921	0.0189	0.3798	4.69	0.0303	−0.1920	0.0889	−0.3662	−0.0178	4.67	0.0307	−0.8711	0.0740	−1.0160	−0.7261	138.66	<,0001
**Quarter**	**4**	0.0000	0.0000	0.0000	0.0000			0.0000	0.0000	0.0000	0.0000			0.0000	0.0000	0.0000	0.0000			0.0000	0.0000	0.0000	0.0000		
**sd**	**24**	−0.3918	0.3177	−1.0144	0.2309	1.52	0.2175	−0.0100	0.1584	−0.3205	0.3006	0.00	0.9498	2.3499	0.1641	2.0283	2.6715	205.12	<,0001	1.0060	0.1311	0.7490	1.2630	58.86	<,0001
**sd**	**25**	0.7790	0.2792	0.2318	1.3263	7.79	0.0053	0.1033	0.1474	−0.1855	0.3922	0.49	0.4832	0.7910	0.1417	0.5134	1.0687	31.18	<,0001	−0.0656	0.1198	−0.3004	0.1691	0.30	0.5836
**sd**	**26**	0.1066	0.2892	−0.4603	0.6734	0.14	0.7125	−0.8588	0.1420	−1.1370	−0.5806	36.60	<,0001	0.0851	0.1400	−0.1893	0.3595	0.37	0.5433	−0.6767	0.1193	−0.9105	−0.4428	32.17	<,0001
**sd**	**27**	−0.4350	0.4905	−1.3963	0.5263	0.79	0.3751	−1.1407	0.2308	−1.5930	−0.6883	24.42	<,0001	−1.6117	0.2407	−2.0835	−1.1399	44.83	<,0001	−1.3705	0.1873	−1.7377	−1.0034	53.52	<,0001
**sd**	**28**	0.0000	0.0000	0.0000	0.0000			0.0000	0.0000	0.0000	0.0000			0.0000	0.0000	0.0000	0.0000			0.0000	0.0000	0.0000	0.0000		
**Depth**	**30**	0.8852	0.2766	0.3430	1.4274	10.24	0.0014	0.8060	0.1447	0.5224	1.0896	31.02	<,0001	1.0065	0.1614	0.6902	1.3228	38.89	<,0001	1.2411	0.1308	0.9848	1.4974	90.08	<,0001
**Depth**	**50**	1.0408	0.2508	0.5492	1.5324	17.22	<,0001	1.5299	0.1266	1.2817	1.7781	145.93	<,0001	1.5963	0.1234	1.3544	1.8381	167.36	<,0001	2.1747	0.1099	1.9593	2.3902	391.31	<,0001
**Depth**	**70**	0.4152	0.2459	−0.0668	0.8972	2.85	0.0913	1.0815	0.1255	0.8355	1.3276	74.24	<,0001	1.6409	0.1233	1.3991	1.8826	177.03	<,0001	2.1186	0.1082	1.9064	2.3307	383.07	<,0001
**Depth**	**90**	0.0000	0.0000	0.0000	0.0000			0.0000	0.0000	0.0000	0.0000			0.0000	0.0000	0.0000	0.0000			0.0000	0.0000	0.0000	0.0000		
**s50cpue1**	**3**	−1.5803	0.2599	−2.0896	−1.0709	36.98	<,0001	−1.7274	0.1409	−2.0036	−1.4512	150.30	<,0001	−1.4568	0.1394	−1.7300	−1.1835	109.18	<,0001	−2.5668	0.1179	−2.7979	−2.3357	473.75	<,0001
**s50cpue1**	**12**	−0.5397	0.2520	−1.0337	−0.0458	4.59	0.0322	−0.7981	0.1489	−1.0900	−0.5062	28.71	<,0001	−0.8068	0.1400	−1.0813	−0.5324	33.20	<,0001	−1.3838	0.1184	−1.6159	−1.1517	136.51	<,0001
**s50cpue1**	**60**	−0.4254	0.1992	−0.8159	−0.0350	4.56	0.0327	−0.3339	0.1146	−0.5585	−0.1093	8.49	0.0036	−0.4038	0.1085	−0.6164	−0.1911	13.85	0.0002	−0.6186	0.0954	−0.8057	−0.4315	42.00	<,0001
**s50cpue1**	**150**	−0.1278	0.2209	−0.5607	0.3051	0.33	0.5629	−0.0360	0.1352	−0.3011	0.2290	0.07	0.7899	−0.1476	0.1272	−0.3969	0.1016	1.35	0.2457	−0.1708	0.1119	−0.3902	0.0486	2.33	0.1272
**s50cpue1**	**250**	0.0000	0.0000	0.0000	0.0000			0.0000	0.0000	0.0000	0.0000			0.0000	0.0000	0.0000	0.0000			0.0000	0.0000	0.0000	0.0000		
**Dispersion**		12.0331	0.8721	10.3238	13.7424			5.6308	0.1653	5.3069	5.9547			5.0507	0.1361	4.7839	5.3175			4.0717	0.0955	3.8845	4.2590		
		Wald Statistics for Type 3 Analysis of Model						Wald Statistics for Type 3 Analysis of Model						Wald Statistics for Type 3 Analysis of Model						Wald Statistics for Type 3 Analysis of Model					
**Year**						68.96	<,0001					242.79	<,0001					386.50	<,0001					343.50	<,0001
**Quarter**						121.33	<,0001					4.69	0.0303					4.67	0.0307					138.66	<,0001
**sd**						42.62	<,0001					113.24	<,0001					387.00	<,0001					270.45	<,0001
**Depth**						21.58	<,0001					157.62	<,0001					208.55	<,0001					501.78	<,0001
**s50cpue1**						40.59	<,0001					185.48	<,0001					121.65	<,0001					579.13	<,0001

Results of density and distribution patterns are shown from several model runs of catch rates for each of the juvenile cod length groups: 0–5 cm (incl. 5 cm): len = 3, 5–10 cm: len = 8, 10–15 cm: len = 13, 15–20 cm: len = 18, i.e. 3–20 cm juvenile cod. Model statistics are given with standard error estimated according to the model estimates, and the Type3 contrast is used for each run. (See also Table S2 in File S1).

The analysis covers all of the cod caught in more than 4,750 hauls for the full revised-design BITS survey time series (2001–12), with a total of 1,560 individuals in the 0–5 cm length group, 25,536 in the 5–10 cm group, 41,042 in the 10–15 cm group, 115,153 in the 15–20 cm group, and more than 1.3 million above 25 cm. The GLM applied to estimate parameters (Eq. 2) and test hypotheses for each length group employed a negative binomial distribution and log (the canonical link function) of the CPUE as a linear function of the parameters tested, i.e., assuming that the logarithm of the mean is linear (GENMOD procedure in the SAS vers. 9.2 statistical software [47], [48]). This allows for inclusion of 0-observations (CPUE rounded up to the nearest integer), i.e., zero catches of cod by length. If the assumption of negative binomially distributed data does not hold, an over-dispersion parameter is estimated. The full model, which defines how the expected catch value (E(CPUE); referred to as the cod density here) by length group depends on the descriptive factors and class variables, is given in Equation 2.
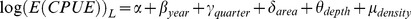
(2)with the model class-level variables including the year, quarter of the year, area (ICES SD), seabed depth and the density of larger cod, above 30 cm in length, as potential predators on juveniles, while the model intercept is 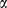. Runs were performed for each individual length group (L) because the model assumes independence between observations, whereas observations for individual length groups are not mutually independent. Plots of residuals versus model-predicted values were produced for each run, and the goodness of fit was checked by comparing the deviance of the full model with the deviance of a version of the model in which the class variables were excluded, i.e., only testing the intercept of the model. The applied GLMs describe the variability in the CPUE data relatively well when considering that binomial models are either fit to 1 or 0, i.e., integers. The models converged, and no trends were observed in the plots of residuals versus model-predicted values. The significant patterns detected in the cod distribution correspond well to previously reported year, quarter, area, and depth variability data found in the literature (e.g., [22], [3], [7]).

The model covers all of the years in the period from 2001–2012, and the seasons tested in the model are the 1^st^ and 4^th^ quarter of the year, following the BITS coverage.

The model stratification according to area was based on the ICES subdivisions (Fig. 1), covering SDs 24, 25, 26, 27, 28 and 29.

The model stratification according to different habitats with different seabed depths covered depths of 0–40 m (incl. 40 m): depth = 30; 40–60 m: depth = 50; 60–80 m: depth = 70; 80–100 m: depth = 90; and >100 m: depth = 110, following the depth stratification used in the BITS survey. A comparative testing was made with an alternative seabed depth stratification using two strata: below and above a 60 m depth.

For a reduced number of observations in the BITS data, where hydrographic data associated with the cod CPUE data were available from the ICES DATRAS database (www.ices.dk; 2003–12), the cod density as a function of the bottom temperature and salinity was tested. Here, the bottom temperature class variable was stratified in 5°C intervals, and the bottom salinity was separated into two classes: Below and above 15 psu (Table S2 in File S1). In this analysis, the bottom depth class variable was omitted, since the bottom hydrographic class variables are correlated with depth.

### Analyses of juvenile cod intraspecific density patterns

The above model (Eq. 2) was also used to investigate how the juvenile cod density patterns depend on the density of co-occurring larger cod, as potential predators on juvenile cod (cannibalism). Here, the mean density of larger cod (>30 cm) was included in the model as an independent variable (s50cpue1) to test for this effect. The density classes employed in this analysis were as follows: 3 (0–5 individuals/haul), 12 (5–20 individuals/haul), 60 (20–100 individuals/haul), 150 (100–200 individuals/haul), and 250 (>200 individuals/haul).

Furthermore, the yearly density patterns for the smallest juvenile cod plotted from the above model as well as the overall yearly distribution area and patterns of the juvenile cod cohorts obtained using the LGCP method (see section below) were compared to the year class strengths of the individual cohorts from 2001–2010. In Table 2, the year class strengths and recruitment of eastern Baltic cod, as estimated by the ICES WGBFAS assessment working group [7] and through BITS indices from the ICES DATRAS database (www.ices.dk), are presented, together with associated information on major North Sea water inflow events in the Baltic Sea basins during the same period according to previous authors [13], [48], [49], [15], [50] and http://www.smhi.se/en/News-archive/improved-oxygen-conditions-in-the-baltic-deep-water-1.21801#bottom.

### Analysing vertical zoning in hydroacoustic and hydrographic data from specialised surveys

**Figure 3:**
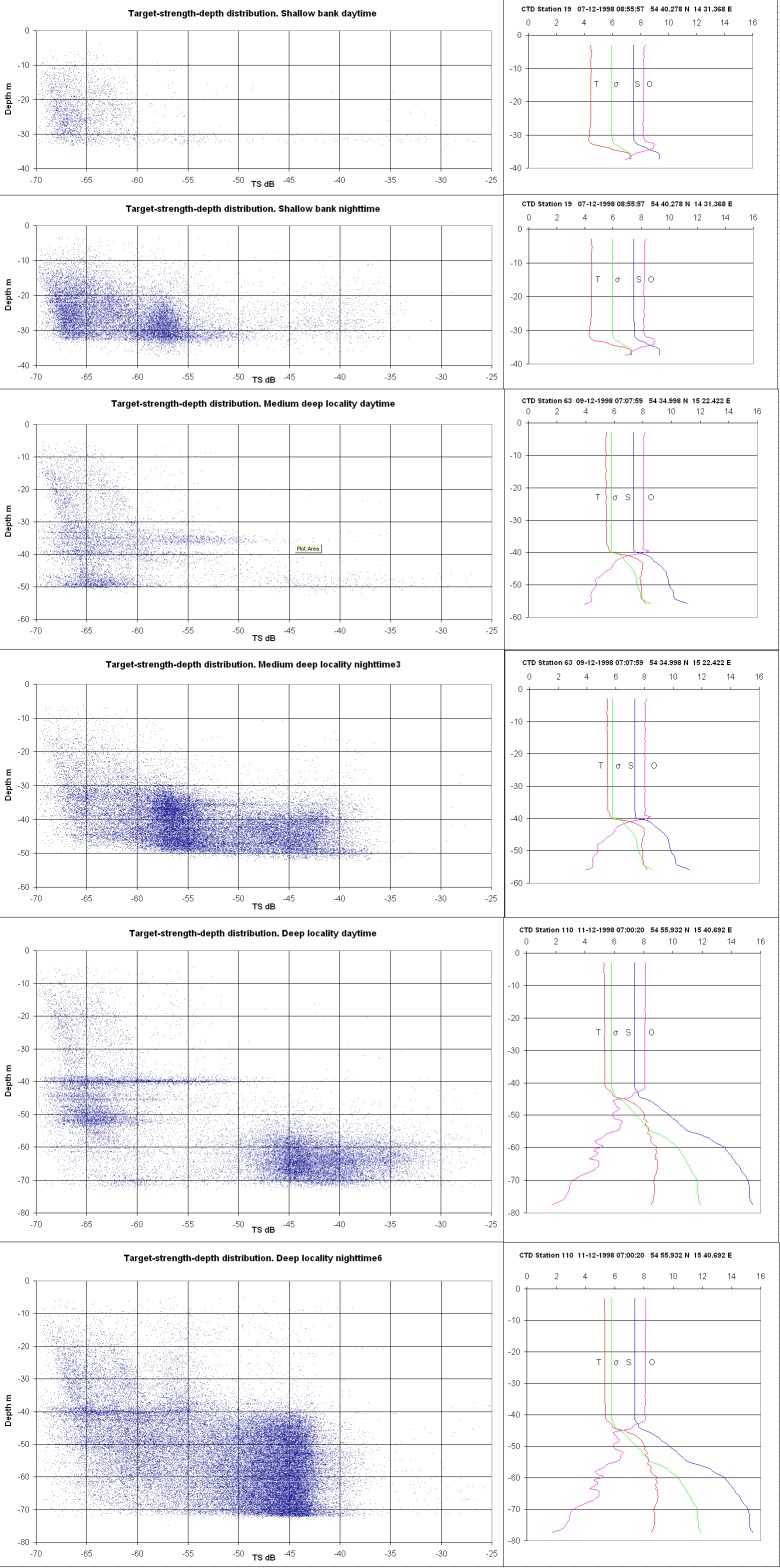
Combined observed TS distributions over 2 full nights and 2 full days continuous recording at each type of locality according to depth, diurnal time, and vertical hydrographical frontal zones as recorded with CTD, i.e. vertical water layer stratification at the 3 types of localities.

**Figure 4:**
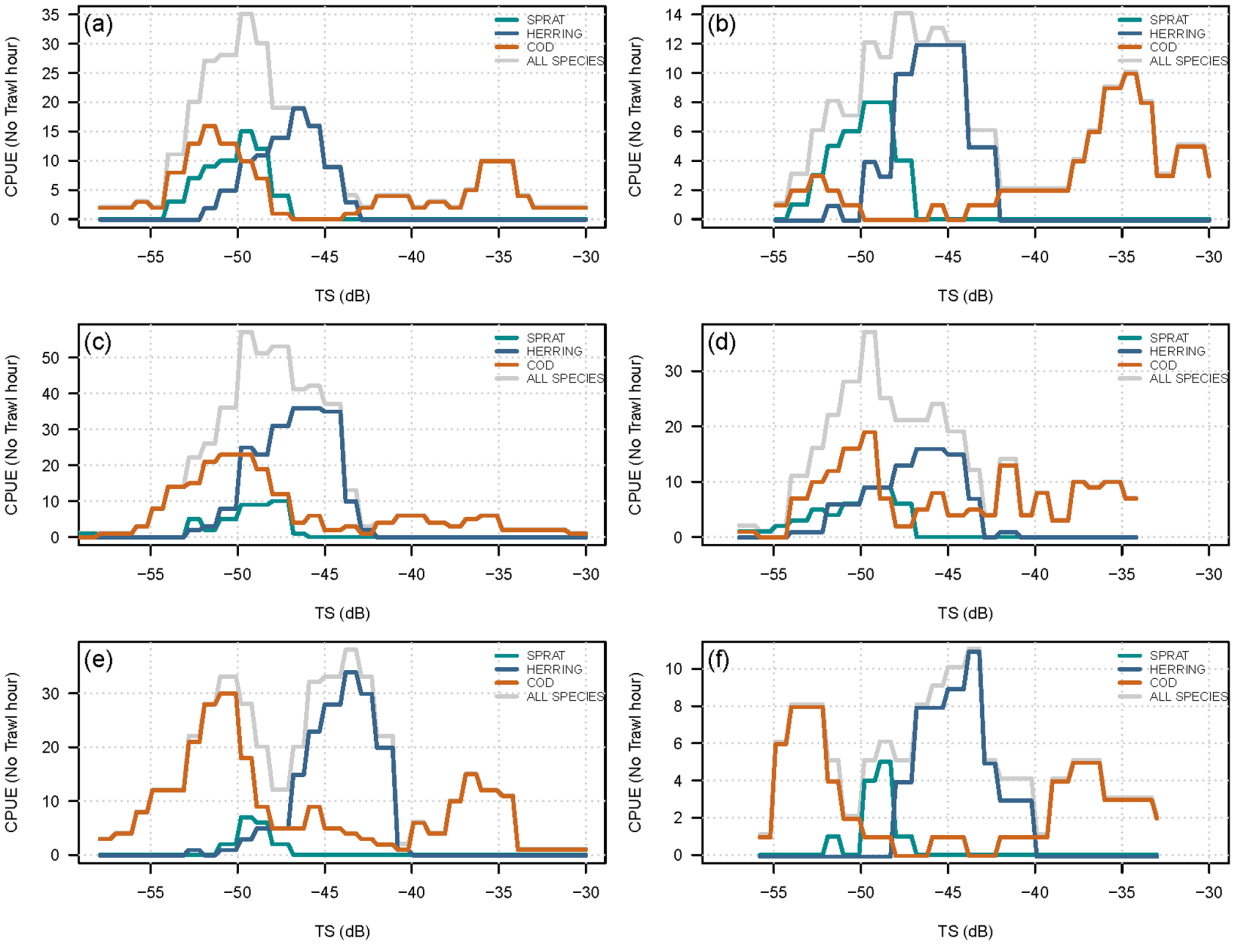
Trawl CPUE of cod, herring and sprat from the special investigated types of localities (Table 1, Fig. 2) during the 1998 specialized surveying according to bottom depth and time of day by length group re-calculated to target strength (TS) distributions. The TS distributions are estimated from observed species specific length distributions in trawl catches at the same stations using the juvenile cod TS-length algorithms from Nielsen and Lundgren (1999) and the clupeoid TS-length algorithm from ICES [8].

Typical distributions of single targets as a function of depth and TS related to vertical hydrographic frontal zones, i.e., water layer stratification recorded with the CTD, at the 3 types of localities are presented in Figure 3. These distributions cover 2 days and 2 nights of continuous acoustic recording at each locality. They are compared to the calculated TS distributions summarised from trawl CPUE data for cod, herring and sprat from these localities from night or day, as plotted in Figure 4. Here, the TS distributions were calculated from the observed (trawl-caught) species-specific length (L) distributions at the same stations using the following TS-length algorithms: Juvenile cod <15 cm: TS = 27log_10_L-76.0 dB [52], [53];

 Cod ≥15 cm: TS = 20log_10_L-67.5 dB [21];

 Sprat and herring: TS = 20log_10_L-71.2 dB [21], [41]

### Cod distribution and density patterns based on the LGCP model

**Figure 5:**
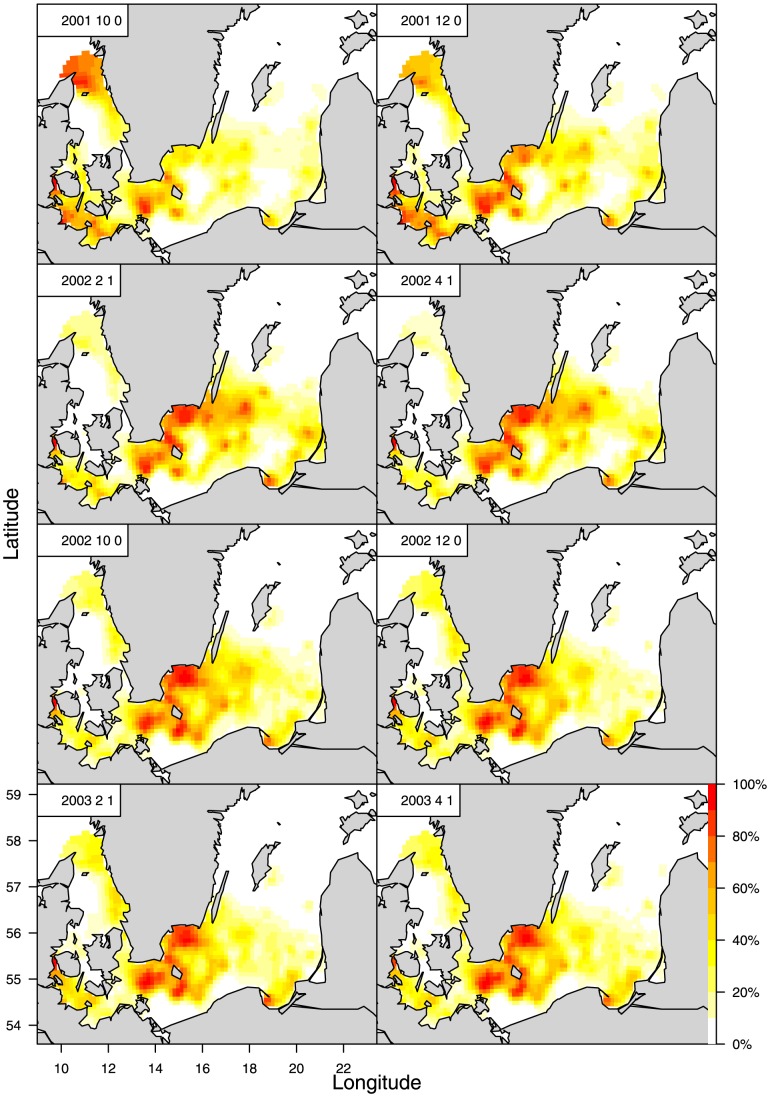
Distribution and density patterns in form of abundance maps of juvenile cod cohorts as 0-group in the autumn and early 1-group in the following spring where 0-group cod in year Y is correlated with 1-group in year Y+1. The abundance maps are estimated from correlation analysis with the LGCP statistical co-variance model (Eq. 3) of BITS data (DATRAS exchange format) for the cohorts 2001–2002.

**Figure 6:**
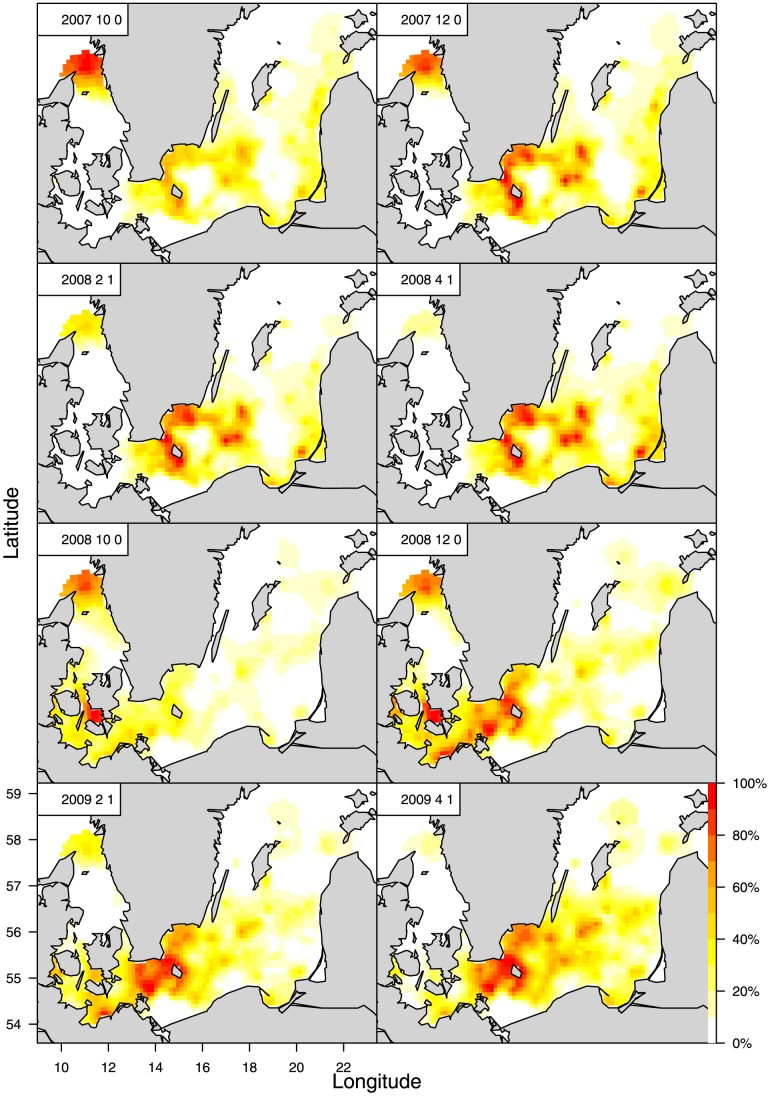
Same as Figure 5, but for the juvenile cohorts 2007–2008.

**Figure 7:**
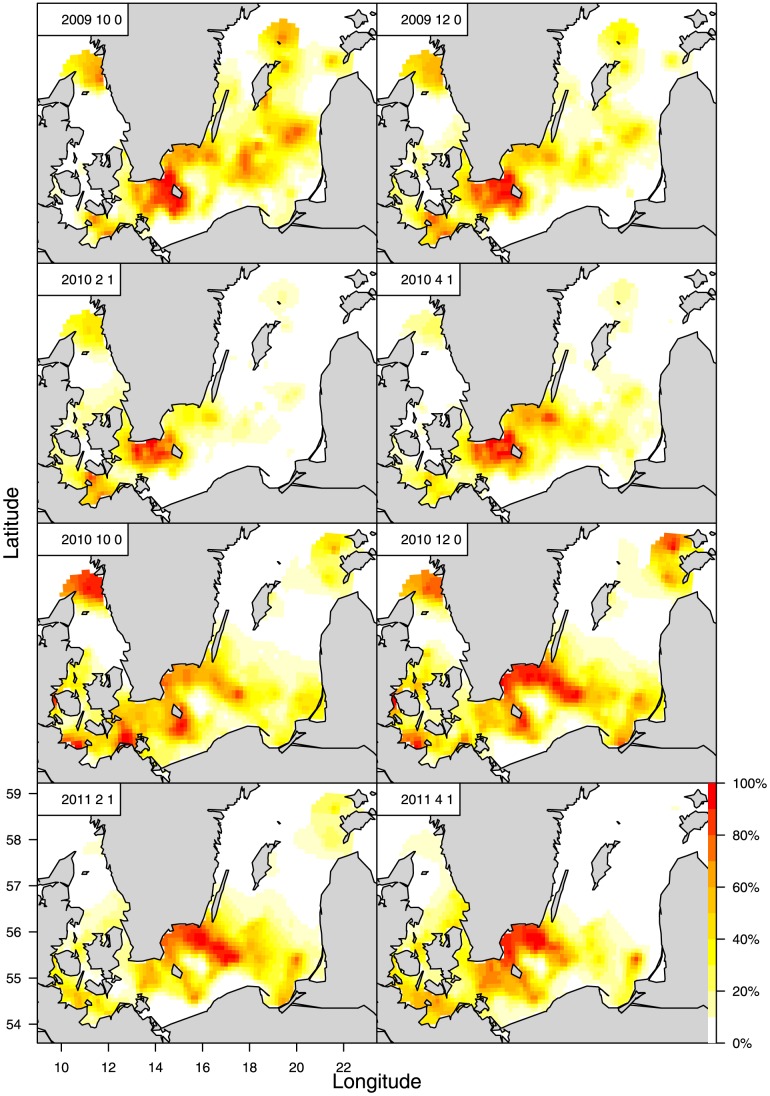
Same as Figure 5 but for the juvenile cod cohorts 2009–2010.

The distribution and density patterns of the juvenile Baltic cod cohorts, the 0-group, in autumn and the early 1-group in the following spring, are shown in animated abundance maps for the years 2001–2011 calculated from the LGCP model with parameters obtained by correlation analysis of BITS data. This model makes unbiased estimates of fish abundances by time and space for 0-groups [34], but in contrast to most survey abundance models, which assume the numbers caught in one haul to be independent of the numbers caught in all other hauls, the LGCP model utilises the positive correlation between the numbers of fish caught when the spatial distance between the hauls decrease. The current model is modified to follow individual cohorts, where the 0-group cod in year Y are correlated with the 1-group in year Y+1. It is advantageous to follow the cohort distribution and movement of the late-spawned 0-group into the next year as the early 1-group to avoid the assumptions about growth rates that would be made for early- and late-spawned juvenile cod, respectively, if only length groups were followed assuming a natural length-based correlation. Hence, the model estimates spatial and seasonal correlations assuming Poisson-distributed observations and multivariate log-normal means, including zero observations and over-dispersion, a spatiotemporal correlation structure and potential correlation between different cohorts. Accordingly, the LGCP model estimates the density 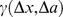 with the co-variance model as follows (Eq. 3):
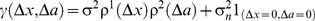
(3)where 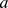 is the fractional cohort age (e.g., for the 2001 year class caught in month 2 of 2002, a = 1.167 years); 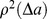 is the age correlation at a separation of 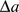; 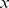 is the position (spatial); 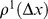 is the spatial correlation at a distance of 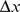; 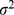 is a variance parameter for large-scale variation; and 
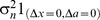
 is the variance for small-scale variation. Samples of highly disaggregated spatial and seasonal gridded maps (3*3 nautical miles, per month) are shown in Figures 5, 6 and 7 (and Figs. S1, S2), as estimated from spatial, seasonal and intra-cohort age correlations in the BITS observations. The LGCP model fits the data and converges well, with a high intraspecific time and spatial correlation, when it is parameterised using the maximum likelihood method and the Laplace approximation, where the maxima and uncertainty can be estimated from the positive definite Hessian matrices in which all rows are independent.

### Ethics statement

No humans or primates or laboratory animals have been involved in the study. There has been no sampling from private land, and the field studies did not involve endangered or protected species. Only fish sampled in public sea areas have been used. All fish have been sampled with research survey trawls under or related to ICES (International Council for Exploration of the Sea; www.ices.dk) coordinated international standard trawl and hydroacoustic surveying. The sampling and handling of fish follows strictly all ICES guidelines, procedures, legislative rules, and permissions from national governments for sampling and handling of fish in fisheries research surveys. The sampling was conducted by national government owned research vessels following Danish national legislation, permissions, and ethics for handling of wild caught fish. The sampling has been performed under repeated international standardized surveying where the research vessels have full permission to sample from all relevant national public authorities (governments) in the Baltic waters.

## Results

### Influence of geographical area and topography (H01 and H02)

The results of the GLM (Table 3) applied to the BITS CPUE data show that the highest densities of the smallest juveniles (0–5 cm) are found in SD25, followed by SD26, corresponding to known spawning areas in the Bornholm Basin and Gdansk Deep. Lowest densities are observed in SD24 and SD27. However, juvenile cod exhibit an increasing density with increasing size in SD24 (Arkona Basin), and for the sizes of 10 cm and larger the highest densities occur in this area. The highest seasonal density of the smallest cod is found in the 4^th^ quarter (Table 3), but they appear only seldom at that time in SD29, while always observed in all areas in the 1^st^ quarter (latter results not shown). Also, juveniles of the 5–10 cm length group are found with the highest densities in SD25, but then followed by SD24 and SD28 (Gotland Basin). In general for all size groups the densities are low in SD27 during 2001–2012. No major seasonal density differences could be detected for the larger size groups, with the exception of the 15–20-cm group showing the (statistically significant) highest density in the 1^st^ quarter.

The above significant patterns in the geographical distribution of nursery grounds are also observed in the high-resolution density patterns resulting from the statistical LGCP model applied to the individual 2001–2010 cod cohorts. (Figs. 5, 6, 7; Figs. S1, S2). Even as small, 0-group juveniles in Oct.–Dec. the cod shows a widespread geographical distribution area in the CBS, ranging from SD22 to SD28. The distribution of this group is generally scattered but also presents high-density concentrations in the central Baltic basins. The main concentrations are found in the Arkona, Bornholm, Gotland, and Gdansk Basins and in the more coastal Hanö Bay (Fig. 1). The same wide distribution and concentrations are estimated for the early 1-group in Feb.–Apr. by the LGCP model; i.e., the modelling indicates a high consistency in the distribution patterns for different seasons and juvenile life stages. However, variation is observed in the extension of the distributions between years, where both the 0- and 1-groups show a very northerly distribution in the later years (2007–2010 cohorts), up into the north and east of Gotland and along the western coasts of Poland, Lithuania, Latvia, and Estonia, which is not observed for the early period cohorts.

### Influence of water layers and seabed depth (H03 and H02)

Juvenile cod were caught at localities with bottom depths ranging from 16 m to more than 100 m, though they occur with a relatively low density at bottom depths deeper than 80 m.

The vertical distributions of juvenile cod found in the specialised surveys were near-bottom and pycnocline-associated (Figs. 3–4). No juvenile cod with sizes of 2–3 cm or larger were caught in the upper and middle pelagic water layers above the pycnocline with any of the small trawl gears used (Table 1; 0 values not shown); i.e., these size classes were not found in V-shaped, double oblique hauls only covering the surface and mid-water layers. Juvenile cod of lengths 3 cm and larger were all trawl caught in the near-pycnocline and seabed layers in bottom hauls, during both day and night (Figs. 3–4), indicating a constant, rather demersal distribution for these life stages. These findings further indicate that settling occurs at a length of approximately 2–3 cm for the central Baltic juvenile cod.

Regarding the interpretation of the vertical TS distributions shown in Figure 3, TS estimates from the literature must be used. Juvenile cod, sprat and small herring exhibit TS values within the same range (Figs. 3, 4; [25]). Nielsen and Lundgren [52] found TS values ranging from −59.8 to −44.8 dB for 0-group cod in the size range of 75–98 mm and from −57.1 to −37.0 dB in the size range of 159–188 mm (North Sea salinities). Nakken and Olsen [54] reported TS values between −50 dB and −47 dB for fixed, anaesthetised juvenile cod in the size group between 7 and 9 cm. Ona [55] estimated a mean TS of −57.1 dB, with a distribution ranging from −69 dB to −48 dB during night time in a rearing pond for juvenile cod in the size class of 3–8 cm (mean length, 5.1 cm). Accordingly, cod in the size range of 3–15 cm mainly show a TS distribution ranging from −60 dB to −45 dB, consistent with Figure 4. Numerous targets and dense layers of mysids were easily detectable and distinct in the hydroacoustic 38 kHz split beam profiles, especially during night time. The plankton species composition in different water layers was investigated via depth-stratified fishery sampling using BONGO and MIK ring trawls during the specialised surveys, which showed dense aggregations of mysids, with the dominant species being *Mysis mixta* [56], [57]. The detected mysids were up to 20 mm in length, and the expected TS values for mysids range from approximately −75 to −65 dB based on the literature [58]. Targets within the range −70 to −65 dB were typically found to be abundant in vertical layers from the sea surface to under the pycnocline (Fig. 3). At localities with well-mixed waters, these targets are more evenly scattered throughout the entire water column. The captured juvenile cod show a TS distribution between −60 and −45 dB (Fig. 3). For this TS range, distinct vertical patterns in the obtained TS distributions and numbers of single fish targets tracked can be observed in the profiles from the three different types of localities, with distinct hydrographic characteristics (Fig. 3). Based on the findings for trawl caught cod and the corresponding fish TS distributions (Figs. 3–4), it appears that in shallow-water bank areas with bottom depths of approximately 40 m, the small-to-medium-sized targets show a more even distribution in the water column starting above the pycnocline (35 m depth) and extending up to a depth of 10 m (Figs. 3–4), while the larger targets of cod and herring are distributed just above the pycnocline, both during night and day. At the intermediate depth localities (60 m) and in the deep basin areas (80 m), the small-to-medium-sized juvenile cod TS is mainly distributed in the pycnocline, where there is extensive stratification related to oxygen, salinity, and temperature, being located around the 30–50 m depth layer in the intermediate depth areas and around the 40–70 m layer in deep sea areas (Figs. 3–4). At deep localities, the density of the water layer below the pycnocline is relatively high. The near-bottom oxygen concentrations vary from near saturation at localities with well-mixed water, showing a continuous decline in the water column beginning at the oxycline at localities with stratified water layers, to very low concentrations (0–1 ml.l^−1^ O_2_) close to the seabed at deep localities. At all types of localities investigated, no targets were detected in the oxygen-depleted layers from the seabed to 5–7 m above the seabed, which corresponded to oxygen concentrations below 7 ml.l^−1^ O_2_ in the shallow bank areas, below 4 ml.l^−1^ O_2_ in the intermediate depth localities, and below 2 ml.l^−1^ O_2_ in the deep basin areas. However, in the deep sea basin areas, most of the targets, including juvenile Baltic cod, were observed in water layers with oxygen concentrations between 2 and 4 ml O_2._l^−1^, regardless of the size group.

Distinct diurnal patterns could be observed in the distribution of the acoustic targets, including those corresponding to juvenile Baltic cod (Figs. 3–4), with the smallest juveniles being found at deep, intermediate depth and shallow localities at both day and night, but with the highest catch rates occurring during night at the intermediate depth and deep localities. Single targets in the TS range of juvenile cod appear to be concentrated during night time in and below the pycnocline, compared to a more scattered distribution during the day, extending well above the pycnocline.

The results of the GLM (Table 3) show distinct trends in the vertical distributions and density patterns of the juvenile cod according to bottom depth stratification. For the smallest juveniles, with lengths of 0–5 cm, the densities are higher at localities with bottom depths of 40–60 m than in shallow areas (0–40 m bottom depth), and the densities decrease significantly with increasing bottom depths from 60 m for this size group. For the larger, 5–10 cm and 10–20 cm juveniles, the highest densities are also observed at localities with a depth of 40–60 m, followed by those with 60–80 m, but with lower densities being recorded in shallow areas (0–40 m depth) for the largest juveniles (10–20 cm). All size groups (0–20 cm) of juvenile cod are found in the deepest areas, with bottom depths greater than 80 m, but with significantly lower density here. In the 4^th^ quarter of the year, the smaller juveniles occur more frequently at depths greater than 80 m compared to the 1^st^ quarter, and in general, juvenile cod are seldom found at localities with bottom depths greater than 100 m (not shown). Accordingly, as the juveniles become larger, there is a tendency toward their densities increasing in the deeper habitats and localities. No differences in density were found due to the near-bottom salinity, but significantly lower densities were observed in bottom waters with a temperature of less than 5°C compared to the 5–10°C and above 10°C temperature strata. This appears to be a generally consistent and significant pattern for all juvenile cod size groups (Table S2 in File S1).

### Influence of year class strength and intraspecific density (H04)

The GLM results show significant variations in the juvenile cod density between the different study years. For the smallest, 0–5 cm juveniles (mainly the 0-group), the highest densities are found in the years 2001, 2003, 2004, 2007, and 2008, and intermediate densities are observed in 2006, while the lowest densities are found in 2002 and from 2009–2012. The densities recorded in 2003, 2004, and 2007 are significantly higher than in 2012, and the highest densities occur in 2004 and 2007. In late spring of 2003, there was a strong inflow to the Baltic, resulting in favourable hydrographic conditions for spawning and cod fry survival; however, the density of the smallest cod was not found to be higher at this time than in the other high-density years, even though the peak spawning period of the eastern Baltic cod stock is during summer [59], [60]. A similar pattern of high densities in 2001, 2003, 2004, and parts of 2007 and 2008 and generally lower densities in the most recent period, from 2009–2011, is observed for larger juveniles, in the 5–10 cm, 10–15 cm and 15–20 cm length groups. Peak densities are observed in 2004 for the larger cod, extending into 2005 for the largest juveniles, which may correspond to the 2003 cohort.

Distinct intraspecific density dependence is indicated by the results from the GLM (Table 3). For all of the length groups of juveniles investigated, there is a significant increase in density associated with an increasing density of large cod with sizes above 30 cm, which most likely means that juveniles and larger cod aggregate in habitats that are favourable or attractive for both groups.

The geographical distribution and density patterns of the juvenile cod vary with the year class strength for eastern Baltic cod. The year classes formed in 1976, 1977, and 1980 were strong due to favourable conditions for reproduction in the spawning areas in the southern and central Baltic Sea [7], which resulted in the highest historical levels of SSB being observed in 1982–1983. These conditions were associated with frequent inflows of oxygenated, saline water from the North Sea. During the period investigated in the present study, from 2001–2011, the 2003, 2006, 2007, 2008, 2009, and 2010 year classes were relatively strong according to the ICES WGBFAS assessment [7], which was partly confirmed by the ICES DATRAS indices (www.ices.dk) for the 2003, 2006, and 2007 year classes (Table 2). In the same period, strong inflow events into the Baltic were only recorded in spring 2003 and autumn 2011 (Table 2). There was a strong year class associated with the 2003 inflow, but other year classes were also relatively strong, even when no major inflow was observed. Overall, there was not complete consistency in the overlap between the years with the highest densities of the smallest juveniles (2001, 2003, 2004, 2007, and 2008 and, to a lesser extent, 2006) and the years with estimated high recruitment (2003, 2006, 2007, 2008, 2009, and 2010). However, given the annual variability in the overall distribution between years observed from LGCP modelling, it is clear that in more recent years, when there have been more frequent relatively strong year classes of eastern Baltic cod (Table 2; [7]), a tendency towards a north-eastward extension of the distribution area for the 2007–10 cohorts of both 0- and 1-group juveniles can be observed.

## Discussion

### Horizontal and vertical distribution and density patterns (H01 and H03)

The distribution and density patterns of juvenile cod have been described in the scientific literature for several sea and coastal water areas, such as the NE and NW North Atlantic (e.g., [61], [62], [63], [64], [65], [66], [67]) and the North Sea (e.g., [68], [69]). However, the distribution patterns of juvenile Baltic cod have only been described theoretically via hydrodynamic modelling, with only limited comparisons being made with survey data and fishery observations (e.g., [22], [3]). In contrast, vertical and horizontal distribution patterns have been investigated for larger, mature and spawning cod based on hydroacoustic surveys ([59], [60].

It appears from the present GLM analyses, that the smallest juvenile Baltic cod (0–5 cm) occur with the highest densities within the known spawning areas in the Bornholm Basin (SD25), Gdansk Deep (SD26) and parts of the Gotland Basin (SD28), while larger juveniles show the highest densities in more westerly areas in the Arkona Basin (SD24), followed by the Bornholm Basin (SD25). The smallest size group is mainly observed during the 4^th^ quarter, corresponding to individuals from the late summer peak spawning period of eastern Baltic cod (SD25, SD26, SD28) [70], [71]. The increasing density associated with increasing size in SD24 could indicate migration between areas, where Eero *et al.* [29] also found indications of the migration of small cod from SD25 to SD24 in later years. Survey trawl gear selectivity associated with differences in the spawning seasons and growth of eastern and western Baltic cod could influence the survey catchability of the smallest juveniles (<2 cn); e.g., the juveniles in SD24 might have grown to a larger size class before being caught in the 4^th^ quarter survey. However, given the early spawning of western Baltic cod in the spring, the smallest juveniles would most likely have been observed in at least small numbers in the 1^st^ quarter surveys in SD24 if they were abundant here. Moreover, catchability effects do not influence the finding that there is a consistently higher density of the larger size groups in westerly areas. The applied LGCP statistical modelling confirmed these overall geographical distribution patterns on a high resolution scale in time and space. Here, it should be noted that the LGCP results are not influenced by gear selectivity to the same extent as the GLM results because the LGCP model adjusts the mean quarterly CPUE values of the cohorts according to the correlations between the quarterly observations. It appears that late 0-group and early 1-group cod are widely distributed throughout the CBS, with the highest concentrations being observed in the basins and the more coastal area of Hanö Bay, which is consistent over seasons for the cohorts. The annual variability shows a clear tendency towards a north-eastward extension of the distribution areas in the more recent years of the investigated period (2007–2010 cohorts). This may be associated with a more frequent occurrence of relatively strong eastern Baltic cod year classes. Consequently, even though the juvenile cod consistently show the highest concentrations in the Baltic basins, without considerable yearly and seasonal variation by age being detected, they are still widely distributed, and the extent of their distribution varies by year; i.e., they do not exhibit geographically limited nursery areas.

Concerning the vertical distribution, the GLM revealed an increasing juvenile cod density associated with bottom depth as the fish become bigger. The main nursery areas for the smallest juveniles are found at bottom depths down to 60 m, with peak densities occurring at 40–60 m, while larger juveniles show the highest densities at depths of 50 to 80 m. However, all size groups are found at localities with bottom depths of greater than 80 m (down to 100 m), but at lower densities, which is consistent with the wide distribution described above. The hydrographic conditions, especially the oxygen concentrations, in the near-bottom water layers appear to have a significant influence on the juvenile Baltic cod distribution and density patterns based on comparison with the results from the integrated trawl and acoustic specialised surveys. Indeed, juvenile cod show the highest abundance in well-oxygenated waters and in waters warmer than 5°C, but they also occur at deeper localities with oxygen-depleted waters, and a great deal of variability is observed between years. Even the smallest size group of juvenile cod is found at deep localities with oxygen-depleted waters, where the bottom oxygen concentrations can fall to 2 ml.l^−1^ O_2_ (or even lower). Juvenile cod are found both at stratified and well-mixed localities and at localities where the distance between the pycnocline and the bottom is rather high. However, at the stratified localities, there is a relatively lower density observed in the near-bottom water layers with oxygen O_2_ concentrations <2 ml.l^−1^. The choice of this reference tolerance limit for testing the occurrence of juvenile Baltic cod is supported by the limits of approximately 2.4 ml.l^−1^ found for cod in the Gulf of St. Lawrence [72] and approximately 3 ml.l^−1^ reported for adult cod in the CBS by Tomkiewiez *et al.* [59]. Similar oxygen tolerance levels for Atlantic cod have been documented by Plante *et al.* [12] and Chabot and Dutil [11], and physiological experiments examining gas secretion and resorption in the swimbladder of juvenile cod related to vertical migration carried out by Harden Jones and Scholes [73] indicate that extensive, long-range diurnal vertical migrations of juvenile cod are possible. Neuenfeldt e*t al.* [13] found that adult Baltic cod could remain for several hours in hypoxic waters showing less than 50% oxygen saturation to forage. We observed that juvenile cod occur in relatively low numbers in the nearest-bottom water layers (up to 5–7 m above seabed), according to the acoustic single-target distributions recorded in both day and night; however, the exact location from the seabed up to approximately 6 m cannot be determined from the trawl fishery sampling conducted here, taking the trawl gaps into account.

The characteristic hydrographic feature of the deep central Baltic basins is a permanent halocline separating an intermediate cold water layer from a saline bottom water layer [1], [2], [13]. Within the deep water and bottom layers (>60 m), oxygen depletion has often been observed, but well-oxygenated water is normally found in the halocline (40–60 m depths) [22]. Simulations of the seasonally averaged drift patterns of cod larvae spawned at different times in the Bornholm Basin from 1986 to 1999 conducted by Hinrichsen *et al.* [22] predict both a northern and southern distribution of settling sites around Bornholm Basin in shallow-water (coastal) areas compared with observed distributions from juvenile pelagic and demersal (BITS) trawl surveys for the period 1993–2000. Here, the densities of juvenile cod were predicted to be highest in southern areas with bottom depths of less than 40 m for early- and late-spawned individuals. A problem in this case is that the settled stages of juvenile cod were not well covered by the BITS survey design for this time period. The results may therefore be flawed due to the different trawl catchability results according to area. Hinrichsen *et al.* [3] conducted the same type of drift model simulation to predict transport patterns for larvae spawned in the three major spawning grounds of the CBS for the period from 1979–2004 to predict potential settling and nursery areas of early juvenile eastern Baltic cod and potential habitats showing a high probability of successful settlement. They concluded that the settling and early nursery areas are situated at the edges of the basin, down to where the halocline meets the bottom, while the probability of settlement in the deeper central parts of the basin is low because of the minimum oxygen requirements for successful settling. This means that settling would only be expected to occur on the northern and southern slopes of Bornholm Basin, the western and eastern slopes of Gotland Basin, the eastern part of the Gdansk Deep, and along the Lithuanian and Latvian coasts, showing both yearly and decadal variability. Concerning oxygen requirements, these authors refer to Chabot and Dutil [11], indicating that environments exhibiting an oxygen saturation below 40% are not suitable for settling, resulting in a reduced probability of successful settlement. Additionally, they refer to the finding that data storage tags indicate that Baltic cod remain in less-oxygenated water masses (<40% oxygen saturation) for feeding purposes only ∼10% of the time. Finally, they note that they did not study the importance of swimming ability when examining the drift of virtual larvae and juvenile cod. When Hinrichsen *et al.* [74] modelled the passive drift of simulated cod eggs and larvae originating from Kattegat to the Eastern Baltic Sea Basins over 80–100 days before settling, it was observed that they could be distributed over a long distance in all directions from nearly all spawning sites before settling. Accordingly, the pelagic fry are most likely distributed over a large potential settling area, limited by various factors, as indicated by other studies addressing the optimal and lethal food and oxygen conditions for settling.

In contrast to the predictions from these studies, we observed that 3 cm and larger juvenile cod are widely distributed throughout the CBS, including in deep sea areas with oxygen saturation well below 40%. Consequently, hypothesis H01 is rejected. The settled stages do not occur only in shallower (more oxygen saturated) areas down to 60 m, e.g., at the edges of the Baltic basins, and show limited geographical nursery areas across years. Hypothesis H03 is also rejected, as the settled stages exhibit distinct vertical distribution patterns according to hydrographic vertical stratification. However, despite concentrating in certain layers according to hydrographic factors and frontal zones, these stages show a high tolerance and are also widespread at deep localities with less suitable hydrographic conditions.

### Potential schooling behaviour of juvenile Baltic cod (H02)

The investigated settled juvenile Baltic cod do not aggregate in dense schools but show a more scattered distribution over a larger area and within the water column. In the North Sea, cod larvae and pelagic 0-group cod are more abundant and show a better condition at frontal zones than found elsewhere at neighbouring sites [69]. Settled juvenile cod have been found to aggregate at the north-eastern edge of Georges Bank at 70–100 m depth dependent on seabed sediment type and hydrographical features [67]. The concentrations of juveniles can also be expected to be associated with hydrographic frontal zones in the Baltic, and we detected the highest concentrations close to the pycnocline frontal zone. Distinct patterns (patchiness) could be observed in the vertical distribution of single zooplankton targets (most likely mysids), which during night time in the late autumn and early winter, were very similar to the single target distribution observed for juvenile cod at localities with both stratified and well-mixed waters. The juvenile cod distribution can be associated with predation on mysids. Mysids were found in the stomach contents of the captured juvenile cod even at very deep localities, though in smaller relative amounts compared to the fish caught in shallower-water localities, where the juvenile cod were found to be in significantly better condition (not shown). This observation is in accordance with the findings of Patokina and Kalinina [75] and Hüssy *et al.* [76], who reported that Baltic cod smaller than 20 cm distributed in bottom depths as low as 50 m were found to feed mainly on mysids (*Mysis mixta*), while at depths of up to 75 m, benthos (Polychaetes) represented the predominant food source. The distribution of pelagic life stages corresponding to metamorphosed juvenile cod 2 cm long and smaller has not yet been fully mapped. The offshore and coastal waters of the CBS have been surveyed intensively in all areas, in all depths and layers, and during all periods of the year. These surveys have been conducted using a broad variety of small-meshed trawl sampling gears and gill nets, including specially designed young fish trawls and ring nets targeting juvenile life stages, in addition to associated intense hydroacoustic recording, both in specially designed surveys and standard Baltic fish surveys. If the smallest and larger juveniles occur in very dense patches, or in high concentrations in slope areas where the pycnocline meets the seabed, they would have been detected, taking into account the international effort and the combined methods used in the search for these fish during the last 15 years. It appears to be unlikely that the smallest stages of juvenile cod consistently occur in dense patches in the nearest-bottom water layers, outside the reach of the applied trawl gears and acoustic recording apparatuses, as they exclusively feed on pelagic plankton such as copepods (and mysids), and rubber discs that exhibit close seabed contact are used in these trawl survey gears, in addition to the fact that the water layers closest to the seabed are oxygen depleted, making continuous occurrence in these layers unlikely. Accordingly, hypothesis H02, stating that settled juvenile Baltic cod aggregate in dense schools and show schooling behaviour, as observed in the North Sea [68], [69], is rejected.

### Survey fishing efficiency and selectivity in relation to the observed distribution patterns

The diurnal patterns observed in the juvenile Baltic cod distribution are distinct, especially at deeper localities. Diurnal variation in the juvenile cod distribution has also been described in other areas, such as the North Sea [77], [78], [79]. It appears from the present observations of trawl-caught cod and the corresponding fish TS distributions that the smallest juveniles are found both at day and night in deep, intermediate depth and shallow localities, but with the highest catch rates being recorded in the first two types of localities during night. The single targets in the TS range of juvenile Baltic cod appear to concentrate during night time in and below the pycnocline frontal zone, compared to the more scattered distribution detected during day, which extends well above the pycnocline. The higher night time catch rates obtained are in accordance with what has been observed for near-coastal north-western juvenile cod [80]. The greater numbers of night-caught juvenile cod could be due to increased catchability at this time, as the juveniles may not escape through mesh as easily in the dark, when the trawl twines are not visible. However, it is questionable if this makes a difference in the intermediate depth and deep localities, where the intensity of daylight is rather low. The single target TS distributions observed in the acoustic data are not influenced by gear selectivity, and it appears to be evident that night time concentrations are higher. The survey trawl does not catch all juvenile cod. The L50 is not documented for the TV3 trawl, but some of the smallest 0-group cod will escape the trawl, either through the mesh or under the bottom gear. We only caught cod from size groups of 2 cm and larger, and the smallest juveniles were infrequent. Engås and Godø [24] reported escape under the gear footrope (bottom gear) when using bobbin bottom gear, but this is considered to be a minor effect here, as the TV3 trawl has rubber disk bottom gear exhibiting close seabed contact. In the present context, where no absolute abundance estimates of juveniles are used, but the relative density and distribution are analysed, the effects of selectivity and different fishing powers dependent on size are considered unimportant. Although the smallest juveniles were not observed and their distribution and density patterns have not been fully mapped, there is no reason to believe that the fishing power and selectivity in the survey trawls will be different between different years, quarters, areas, or depth strata, thus influencing the results of the present analyses.

### Density dependence in relation to cannibalism and year class strength (H04)

The juvenile stages of demersal fish stocks and the year-class strength are thought to be regulated in part through density-dependent processes including competition for limiting food resources and predation [81], [63], [82]. Cannibalism on juvenile eastern Baltic cod has been documented [26], [83], [27], [28], [18], [29]. The present analyses of intraspecific density patterns indicate that there is a high degree of overlap between juvenile and larger (>30 cm) Baltic cod. Accordingly, the juveniles and the larger cod aggregate in the same habitats, which are favourable and attractive for both small and larger cod. A potential explanation for this phenomenon is that the larger predators seek the habitats of the juveniles to prey on them. However, this does not appear to be a likely overall strategy for the cod, given that investigations of Baltic cod stomach contents conducted in recent years have not indicated any important cannibalism during the investigated period [17]. Furthermore, LGCP modeling showed a north-eastward extension of the juvenile cod distribution area in years with relatively stronger eastern Baltic cod year classes. Accordingly, hypothesis H04 cannot be rejected based on the present data.

### Future studies

Future studies could analyse correlations in the density and distribution patterns of juvenile cod in detail according to the specific distribution patterns of other species such as mysids, sprat and herring, taking into account detailed data on oxygen contents close to the seabed. Here, it is relevant to evaluate the extent of overlap with other potential prey species for larger cod, such as sprat and herring. An obvious tool to be applied in these investigations is LGCP models integrating the correlations between species distributions.

## Supporting Information

File S1 Table S1 (containing acoustic echosounder and hydrographical CTD profiler calibration parameters); Table S2 (containing the generalized linear model maximum likelihood parameter estimates of CPUE for the statistical model 1 in Equation 2 modified to only include the dependent variables year, quarter, salinity and temperature.
(DOCX)

Figure S1 **Same as** **Figures 5** **–** **7** **, but for the cohorts 2003–2004.**
(EPS)

Figure S2 **Same as** **Figures 5** **–** **7** **, but for the cohorts 2005–2006.**
(EPS)
