# Correction: Localisation of Nursery Areas Based on Comparative Analyses of the Horizontal and Vertical Distribution Patterns of Juvenile Baltic Cod *(Gadus morhua)*

**DOI:** 10.1371/journal.pone.0148721

**Published:** 2016-02-03

**Authors:** J. Rasmus Nielsen, Bo Lundgren, Kasper Kristensen, Francois Bastardie

The following information is missing from the Acknowledgements section: The research leading to these results has partly received funding from the EU-FP7-SOCIOEC project under grant agreement No. 289192.
