# Supplementary material for: Localisation of Nursery Areas Based on Comparative Analyses of the Horizontal and Vertical Distribution Patterns of Juvenile Baltic Cod (Gadus morhua)
Source: PLoS One. 2013 Aug 14;8(8):e70668. doi: 10.1371/journal.pone.0070668 (PMC3743789; doi:10.1371/journal.pone.0070668)
Supplement: File S1 — Table S1 (containing acoustic echosounder and hydrographical CTD profiler calibration parameters); Table S2 (containing the generalized linear model maximum likelihood parameter estimates of CPUE for the statistical model 1 in Equation 2 modified to only include the dependent variables year, quarter, salinity and temperature. (DOCX) [file pone.0070668.s001.docx]

**File S1: Supporting Information and Supplementary Material**

Table S1. Calibration parameters and parameter settings for the SIMRAD EY500 Vers. 5.0 mobile hydroacoustic echosounder system at the surveys DS1698, DS0197, and DS1295, as well as for the SEABIRD SBE 911+ CTD.

Table S2. Generalized linear model maximum likelihood parameter estimates of CPUE by using the SAS GENMOD Procedure for the statistical model 1 (Eq. 2), modified to only include the dependent variables year, quarter, salinity and temperature. Bottom depth is not included as this is correlated to salinity and temperature. The negative binomial dispersion parameter estimated by maximum likelihood. Results of density and distribution patterns are shown from several model runs of catch rates for each of the juvenile cod length groups: 0-5 cm (incl. 5 cm): len=3, 5-10 cm: len=8, 10-15 cm: len=13, and 15-20 cm: len=18. The bottom temperature variable is stratified as 2=(<=5˚C), 7=(5-10˚C); 15=(>10˚C), and bottom salinity is stratified as 10=(<=15 psu) and 20=(>15 psu). Model statistics are given with standard error estimated according to the model estimates, and the Type3 contrast is used for each run.
